# Acute appendicitis following the COVID-19 vaccine

**DOI:** 10.1093/jscr/rjac295

**Published:** 2022-06-22

**Authors:** Ani Oganesyan, Michal Schäfer, Caitlyn Lesh

**Affiliations:** Department of Surgery, University of Colorado School of Medicine, Anschutz Medical Campus, Aurora, CO, USA; Department of Surgery, University of Colorado School of Medicine, Anschutz Medical Campus, Aurora, CO, USA; Department of Surgery, University of Colorado School of Medicine, Anschutz Medical Campus, Aurora, CO, USA

## Abstract

We report the case of a previously healthy 69-year-old female who developed appendicitis after receiving the third dose of the Pfizer-BioNTech Coronavirus Disease 2019 (COVID-19) vaccine; no other triggers were identified. We speculate that an association exists which may be mediated by colonic lymphoid hyperplasia, a condition that might be indicative of an enhanced immunological mucosal response to antigenic stimulation. As widespread vaccination coverage continues, it is crucial to monitor and accurately report the adverse reactions that may otherwise remain unidentified in vaccination trials. Therefore, we suggest that adults experiencing spontaneous, severe abdominal pain following COVID-19 vaccination may benefit from seeking emergent medical care. Likewise, providers should have a low threshold to consider and evaluate patients for appendicitis. If a true causal link is identified, the risk must also be deliberated in context with the millions of patients who have been safely vaccinated and the known morbidity and mortality from COVID-19 infection.

## INTRODUCTION

The Coronavirus Disease 2019 (COVID-19) pandemic has led to the rapid development of COVID-19 vaccines [1]. Pfizer-BioNTech and Moderna vaccine subtypes were developed on the basis of modified messenger ribonucleic acid that encodes the SARS-CoV-2 ‘spike protein’, a critical virulence protein located on the outer surface of the viral particle [2]. Commonly reported side effects of the COVID-19 vaccine include fatigue, myalgia, headache and local irritation; however, less commonly reported adverse reactions, such as appendicitis, have been identified and may warrant further investigation.

Acute appendicitis is the most common surgical emergency [3]. The main causes of acute appendicitis are luminal obstruction by fecalith, lymphoid hyperplasia, impacted stool or neoplasm [4]. Obstruction can result in high luminal pressures that alter mucosal integrity, leading to bacterial infection. Infections are thought to contribute to appendicitis by stimulating lymphoid hyperplasia, which results in further obstruction of the appendix. Similarly, many viral disorders are associated with lymph node enlargement as well as compromised appendiceal lumen patency [5]. We describe a case of acute appendicitis post-third dose of the Pfizer-BioNTech vaccine.

## CASE REPORT

A previously healthy 69-year-old female presented to the emergency department with a chief complaint of severe right lower quadrant (RLQ) pain. The patient received her third dose of the Pfizer COVID-19 vaccine ~67 h prior to her presentation. Approximately, 12 h after vaccination, the patient developed myalgias and fever, which gradually resolved within 48 h. The patient then developed lower abdominal pain ~24 h after receiving the vaccine, which migrated to the RLQ and increased in severity. A complete review of systems was negative for signs or symptoms of gastrointestinal illness in the weeks leading up to this event.

On arrival to the ED, vital signs included a temperature of 36.7°C, heart rate of 107 beats/min, blood pressure of 96/61 mmHg, respiratory rate of 18 and pulse oximetry of 93% on room air. ROS was notable for RLQ abdominal pain ongoing for the last several days. Physical exam was concerning for RLQ pain with rebound tenderness. The remaining physical examination was within normal limits, including normal cardiac and respiratory exam and normal electrocardiogram. Additionally, CBC evaluation was notable for a decreased red blood cell count (3.73^*^10^12/l), decreased hemoglobin (11.5 g/dl), decreased hematocrit (34.8%) and increased neutrophils (7.6^*^10^9/l). Creatinine was elevated to 1.17 mg/dl, BUN was elevated to 21 mg/dl and estimated glomerular filtration rate was 48 ml/min/1.73 m^2^. Rapid COVID-19 test was negative. Computed tomography findings demonstrated perforated acute appendicitis with appendicolith visualized at the base of the appendix with associated mesenteric fat stranding, multiloculated free fluid and trace-free air. General surgery consultation confirmed the diagnosis of perforated appendicitis, and the patient was admitted to the general surgery service and was treated with IV ceftriaxone and metronidazole in preparation for immediate surgery. The patient underwent a laparoscopic appendectomy which was converted to ileocecal resection due to disease severity. Following surgery, the patient was treated with prophylactic aspirin, ceftriaxone (IV) and metronidazole. She was discharged home on hospital day 6 in a medically stable condition.

## DISCUSSION

The present study reports an adult patient presenting with acute appendicitis within several days of receiving the third dose of the COVID-19 vaccine. A previous publication by Mitchell *et al*. [6] reviewed cases of appendicitis following administration of COVID-19 vaccines reported to VigiBase, the World Health Organization database of individual case safety reports (ICSRs). They sought to identify relevant cases of appendicitis with COVID-19 vaccines as the suspected medicinal product from spontaneous reports. The 334 unique ICSRs were clinically reviewed, 90% of which were marked as serious, thereby either causing or prolonging hospitalization, with a time-to-onset of appendicitis of 0–4 days, which is consistent with the presentation of the patient described in our report. They proposed that the hypothesis linking appendicitis with COVID-19 vaccines is a robust Th1 immune response, with a potential dose–response relationship. It remains unclear whether appendicitis may occur as a complication of the SARS-CoV-2 vaccine through similar proposed mechanisms related to the inflammation associated with viral entry or reactive lymphoid hyperplasia causing luminal obstruction.

Infectious processes associated with multi-system inflammation have been linked with appendicitis; for example, acute appendicitis is known to have an association with Kawasaki disease and can be secondary to appendicular artery vasculitis. In Kawasaki disease, abdominal features may represent a higher disease severity [7]. While the disease process in our patient appears to be related to this vaccine, it has only been previously reported as it relates to COVID infection, and we highlight that the current evidence might support a causal relationship secondary to timing.

While awaiting further data from the CDC, several implications may be considered when taking this case and recent literature reports into account. First, while abdominal pain usually has a benign etiology in adult populations, as more adults become vaccinated against COVID-19, patients experiencing abdominal pain following COVID-19 vaccination may consider seeking emergent evaluation. Additionally, health care providers should have a low threshold to consider and evaluate for appendicitis among patients presenting with RLQ pain in the post-vaccine period, particularly within the first 28 days [8]. New and emerging cases such as this can also help illustrate the important role of emergency medicine providers in identifying, treating and reporting vaccine-related outcomes. It is equally important that appendicitis and other adverse reactions occurring after COVID-19 vaccination are reported to vaccine manufacturers and governing vaccine event reporting systems as we continue to monitor for more rare but serious side effects that remained unidentified in vaccination trials.

## CONCLUSIONS

Further data are required to better understand the potential association between COVID-19 vaccines and appendicitis as well as proper follow-up to better characterize prognosis and sequelae. If a true causal link is identified, it must also be viewed in context with the millions of patients who have been safely vaccinated and the known morbidity and mortality from COVID-19 infection.

## CONFLICT OF INTEREST STATEMENT

None declared.

## FUNDING

This research did not receive any specific grant from funding agencies in the public, commercial or not-for-profit sectors.

## PATIENT CONSENT

Written and verbal informed consent was obtained from the patient for publication of this case report and accompanying images. A copy of the written consent is available for review by the editor-in-chief of this journal on request.
